# COVID-19 health certification reduces outgroup bias: evidence from a conjoint experiment in Japan

**DOI:** 10.1057/s41599-022-01324-z

**Published:** 2022-09-09

**Authors:** Yoshiaki Kubo, Isamu Okada

**Affiliations:** 1 Department of Law, Politics, and International Relations, Faculty of Humanities and Social Sciences, University of the Ryukyus, Nishihara, Okinawa Japan; 2 Research Institute for Humanity and Nature, Kyoto, Japan; 3 Department of East Asian Languages and Cultures, Hamilton Lugar School of Global and International Studies, Indiana University, Bloomington, IN USA; 4 Program on U.S.-Japan Relations, Weatherhead Center for International Affairs, Harvard University, Cambridge, MA USA; 5 Department of International Development and Cooperation Studies, Graduate School of International Development, Nagoya University, Nagoya, Japan; 6 Present Address: Department of Urban Studies, School of Policy Studies, Kwansei Gakuin University, Sanda, Hyogo Japan

**Keywords:** Psychology, Science, technology and society, Politics and international relations

## Abstract

The psychological theory argues that serious threats cause negative attitudes from ingroups to outgroups. However, the factors that can reduce such outgroup bias caused by the health threats of a pandemic are unknown. Here, we provide evidence that health certifications to prove immunity or negative test result for COVID-19 reduce outgroup bias. Using a discrete choice experiment with a randomized conjoint design in Japan, we investigated public attitudes towards inbound travelers entering the country, including foreigners, immigrants, and tourists. We found that travelers carrying a vaccination certificate or a negative test result for COVID-19 have a higher probability or rating of being admitted to the country. These effects are the same size as those for travelers undergoing self-isolation. Thus, our results demonstrate that health certification can mitigate outgroup bias among ingroup members experiencing threats to health due to the COVID-19 pandemic. We anticipate that the findings would support the combined usage of vaccine passports and negative certificates to reopen the international borders.

## Introduction

The psychological theory argues that serious threats cause negative attitudes in ingroup members towards outgroup members, known as *outgroup bias*, resulting from outgroup derogation or ingroup favoritism (Demirtaş-Madran, 2020). Outgroup bias in anxiety-provoking situations has also been discussed in the case of infection threats (Schaller and Park, 2011). In the COVID-19 pandemic, health threats provoked outgroup bias worldwide (Ahmed et al., 2021; Ahuja et al., 2020; Biswas et al., 2021; Creţan and Light, 2020; Croucher et al., 2021; Dhanani and Franz, 2021; Haokip, 2021; Hartman et al., 2021; Islam et al., 2021; Kock et al., 2020; Moran et al., 2021; Reny and Barreto, 2022; Sorokowski et al., 2020). However, factors that can reduce such outgroup bias during a pandemic have not been sufficiently studied.

This study investigates the factors that may reduce outgroup bias caused by health threats from the COVID-19 pandemic. Outgroup bias is defined here as the negative bias that people in a host country may hold against inbound travelers from abroad, including foreigners, immigrants, and tourists. We focuses on the effect of health certification, that is, a certificate of having been vaccinated against the virus or a certificate of having tested negative for it, in reducing the outgroup bias. Several governments have started issuing and using vaccine passports to balance infection controls and border controls. However, such policy decisions have room to be challenged on scientific, ethical, and legal fronts (Brazal, 2021; Drury et al., 2021; Hall and Studdert, 2021; Pavli and Maltezou, 2021; Tanner and Flood, 2021). One issue of concern is the potential inequality between the holders and non-holders of vaccine passports. An alternative solution to prevent such inequality is to allow COVID-19 negative test reports to travelers who have a reason not to take the COVID-19 vaccine. Thus, we will test the plausible effects of these health certificates in reducing outgroup bias to add an important factor to consider in policy decisions.

This study contributes to accumulating research on the effectiveness of measures to mitigate health threats and thereby decrease outgroup bias. A recent article reviewing studies on xenophobia and anti-immigrant attitudes during COVID-19 argues that ‘we would be remiss not to discuss the pressing need to consider how to best counteract potential xenophobia and anti-immigrant attitudes resulting from the pandemic’ (Esses and Hamilton, 2021). It lists the effects of political discourse, accurate information about racial inequalities, and virtual intergroup contact on xenophobia and negative attitudes towards immigrants as future research agendas. The current study stresses the effectiveness of health certificates in reducing outgroup bias and expands its implication beyond the previous studies.

The article is structured into six sections. In the next section, we review the related literature, followed by the sections on methodology, results, discussion, and conclusion.

## Literature review

Echoing main psychological theories (Demirtaş-Madran, 2020), the behavioral immune system (BIS) theory claims a chain reaction from infectious threats to outgroup bias as the human defense against pathogens (Schaller and Park, 2011). Specifically, negative attitudes towards outgroups are associated with perceived vulnerability to disease, particularly germ aversion and disgust sensitivity (Aarøe et al., 2017; Brenner and Inbar, 2015; Brown et al., 2019; Duncan et al., 2009; Faulkner et al., 2004; Green et al., 2010; Hodson et al., 2013; Hodson and Costello, 2007; Huang et al., 2011; Ji et al., 2019; Kam, 2019; Navarrete and Fessler, 2006; Terrizzi et al., 2013; Zakrzewska et al., 2019). In the context of the COVID-19 pandemic, previous studies have demonstrated outgroup bias against racial/ethnic groups (Ahuja et al., 2020; Biswas et al., 2021; Creţan and Light, 2020; Croucher et al., 2021; Dhanani and Franz, 2021; Haokip, 2021; Islam et al., 2021; Reny and Barreto, 2022; Sorokowski et al., 2020), immigrants (Ahmed et al., 2021; Hartman et al., 2021; Moran et al., 2021), and tourists (Kock et al., 2020), as predicted by psychological theories.

Whereas many studies have focused on increased outgroup bias due to the perceived threat of infection, few studies have investigated the factors that reduce outgroup bias during a pandemic. However, the three studies reviewed below base their arguments on the BIS theory, are most closely related to the question under investigation here, and mainly examine social aspects affecting health threats perceived by ingroup members. First, a study using a social survey in the US regarding Ebola reported that high individualism and low collectivism conditioned the effect of perceived vulnerability to Ebola on xenophobic attitudes (Kim et al., 2016). Second, an experimental study revealed that people tended to avoid immigrants in the US because they did not believe the immigrants shared local norms (Karinen et al., 2019). Third, an experimental study in the US and India found that people are motivated to avoid individuals with high pathogen risk irrespective of group membership, which is contrary of the traditional account of BIS positing that the system is motivated to prevent correlates of pathogens (van Leeuwen and Petersen, 2018).

Against this backdrop, we examine the effect of *health certification* on mitigating outgroup bias caused by health threats perceived by ingroup members. Health certification has been used for safer and greater access to various activities worldwide by reducing infectious threats to public health. As reviewed by an article on COVID-19, several studies have investigated public and behavioral responses to the COVID-19 health certification (Drury et al., 2021). Nonetheless, previous studies have not investigated the impact of health certificates on reducing outgroup bias, which is associated with health threats for ingroup members. We predict that health certification would reduce outgroup bias based on group membership by mitigating infection threat perceptions for the individuals who hold health certificates. This prediction is theoretically consistent with studies on the BIS theory focused on social aspects, but these studies have not tested the effect of health certification. Therefore, this study investigates the impact of health certification in reducing outgroup bias.

## Methodology

### Inbound travelers from abroad as an outgroup during a pandemic

This study uses data from a pre-registered survey conducted in Japan in February 2021, which planned to study public attitudes in the host country towards inbound travelers from abroad during the COVID-19 pandemic. Travel restrictions are effective at the early stages of a pandemic to control interregional spread of infection (Cacciapaglia and Sannino, 2020; Chinazzi et al., 2020; Haug et al., 2020; Linka et al., 2020; Wells et al., 2020). Meanwhile, residents are more likely to be reluctant to accept people entering the region from outside to lower the health threats at the individual level. For example, in the case of international travel, people in the host country tend to have negative attitudes towards people entering their country from abroad. Thus, inbound travelers from abroad become an outgroup for people in the host country during a pandemic.

However, inbound travelers from abroad are not a unitary group. Instead, people with different attributes enter the country from other regions worldwide. For this study focusing on international travel, there are two critical questions. First, do differences in inbound travelers’ nationality cause different responses in the attitudes of host country residents? If so, we hypothesize that people in the host country are less likely to accept a foreigner’s entry than the entry of someone from their nation due to outgroup bias. Second, does health certification mitigate that outgroup bias of people in the host country? If so, we hypothesize that travelers with health certifications are more likely to be accepted by people in the host country regardless of nationality.

### The case of Japan

This study was conducted in Japan, which is an excellent case for three reasons. First, previous studies have repeatedly pointed out that a large proportion of the Japanese population is averse to foreigners (Richey, 2010). Thus, it allows us to assume that the health threats posed by COVID-19 caused outgroup bias against inbound travelers among host residents. Second, the government was initially reluctant to use COVID-19 vaccine passports to shorten the self-isolation days when travelers enter Japan, but started it on 1 October 2021, more than seven months after our survey was conducted (see Supplementary Note 1). Therefore, at the point of our survey, it was difficult to predict the effectiveness of vaccine passports in reducing outgroup bias. Finally, during the COVID-19 pandemic, travel restrictions have repeatedly changed, tightening several times for infection control and easing fully or partially. This has happened not only for economic rehabilitation but also for hosting the 2020 Tokyo Olympic/Paralympic Games in Tokyo (see Supplementary Note 1). Since our survey was conducted during the preparation period of the Olympics/Paralympics, Japan serves as a crucial case to provide lessons to countries hosting mega international events hereafter.

### Experimental design

The current study adopted a population-based discrete choice experiment with a randomized conjoint design, that is, *conjoint experiment*. Conjoint analysis was initially developed in marketing research (Cattin and Wittink, 1982; Green et al., 2001; Green and Rao, 1971; Green and Srinivasan, 1990), but later expanded as a causal inference method with randomized designs in political science (Clayton et al., 2021; Cuesta et al., 2021; Hainmueller et al., 2014; Leeper et al., 2020). Among the conjoint analysis studies, an experiment on public preferences to admit immigration to the US (Hainmueller and Hopkins, 2015) inspired this study to analyze public attitudes about inbound travelers’ entry into the host country.

The survey showed the participants two hypothetical profiles of inbound travelers and asked them about their entry into Japan. The pre-registration planned to examine the types of travelers from abroad who were less likely to be admitted and the types of people in the host country who were less likely to allow admission of travelers from abroad. A traveler was defined as any person who traveled regardless of purpose (e.g., tourism, business, study, and immigration). For details of the pre-registration, see Supplementary Note 2.

Regarding travelers’ characteristics, we predicted that people in the host country would be less likely to admit entry to inbound travelers having a foreign nationality, with inadequate quarantines or without a proper health certificate, from regions with a high spread of infection, and for a short stay. Thus, we set ten attributes to formulate the profiles: *Nationality*, *Quarantine_Certificate*, *Region*, *Duration*, *Purpose*, *Sex*, *Age*, *Speaking Japanese*, *Education*, and *Income*. Supplementary Table 1 shows the translated version of the conjoint design, consisting of 864,000 (= 3 × 6 × 5 × 5 × 5 × 2 × 4 × 3 × 4 × 4) combinations. The survey included two tasks of a paired conjoint experiment for each respondent. When formulating profiles, the program randomized the levels for each attribute and the order of attributes, except for specific combinations, shown in the notes below Supplementary Table 1, for the ease of respondents to understand the profiles. The reason for choosing the attributes are explained below.

*Nationality* has three levels. Besides Japanese nationals, foreigners were divided into two types based on permanent residency to examine the effect of nationality. It reflected the policy change by the Japanese government in September 2020, which enabled foreigners with permanent resident status to re-enter. We predicted that the admission probability or rating is the highest for Japanese nationals, followed by foreigners with residential status in Japan, and the lowest for those without residential status. The difference between foreigners with residential status and Japanese was estimated as the effect of foreign nationality, which indicated outgroup bias due to travelers’ nationality.

Regarding the attribute of *Quarantine_Certificate*, Japan has implemented self-isolation, submitting a moving plan, submitting a negative test report for COVID-19, and submitting a vaccination certificate as measures. Self-isolation is the most reliable way for virus carriers to avoid contact with others during incubation. In contrast, moving plans do not guarantee an absence of contact with others, and negative certificates are not free from false negatives. However, overall, these measures contribute to reducing the probability of virus carriers’ contact with others. Furthermore, Japan started the COVID-19 vaccination program on 17 February 2021, and on 26 July 2021, the government started issuing vaccination certificates for outbound travel. On the other hand, for inbound travelers, Japan had not used vaccination status as an admission criterion or for quarantine until 1 October 2021. Therefore, at the point of our survey, we planned to hypothetically examine the effect of vaccination certificates following the international debates on vaccine passports as described in the literature review. Statistically, the results of these quarantines were estimated as the difference between “Nothing” and other levels.

*Region* includes two levels: regions with widespread infection and those without widespread infection. China and South Korea experienced the spread of infection in the early stages; the US, the UK, and Brazil experienced outbreaks in the later stages. On the other hand, Taiwan had not experienced notable widespread infection until February 2021, when we conducted our survey. Therefore, the negative effect of widespread infection on entry was estimated as the difference between Taiwan and other regions.

In terms of *Duration*, the minimum level was set as one month, considering 14 days self-isolation required by the Japanese government. We predicted that a more extended stay would raise the admission probability or rating.

*Purpose* is a unique attribute in the context of Japan as it hosted the Tokyo Olympics/Paralympics. The analysis explored the likelihood of admission of travelers entering for the Tokyo Olympics/Paralympics.

The other five attributes consider related factors. *Sex* and *Age* take into account the biological characteristics of inbound travelers who are prone to develop severe health conditions related to COVID-19. A meta-analysis analyzing the data of COVID-19 patients until March 20, 2020, reported that males and those aged over 65 years had a greater risk of developing severe conditions from contracting COVID-19 (Zheng et al., 2020). Therefore, they might be less likely to be admitted to the host country due to their higher risk of developing serious COVID-19. Lastly, *Speaking Japanese*, *Education*, and *Income* are included to re-examine the previous studies about public attitudes towards immigrants (Ceobanu and Escandell, 2010; Esses, 2021; Hainmueller and Hopkins, 2014). We hypothesized that inbound travelers with lower Japanese proficiency, lower educational levels, or lower-income levels were less likely to be admitted.

Among these attributes, this study mainly focuses on analyzing the effects of *Nationality* and *Quarantine_Certificate* despite the pre-registered plan. First, regarding *Nationality*, the difference between travelers with the same nationality and travelers with foreign nationality will indicate the outgroup bias during the COVID-19 pandemic. Second, if the interaction between *Quarantine_Certificate* and *Nationality* shows no difference between travelers with the same nationality and those with a foreign nationality, it will reveal the effect of health certification in reducing outgroup bias during the pandemic.

### Survey procedure

We conducted the pre-registered survey in Japan during February 22–24, 2021, when the second State of Emergency had been implemented in Tokyo, Kanagawa, Saitama, and Chiba. Participants were recruited online from registered monitors in a survey firm, Rakuten Insight Inc., through quota sampling to ensure national representativeness by gender, age, and regions (prefectures). The survey planned to collect 4000 observations (= 1000 respondents × 2 profiles × 2 tasks). We conducted a power analysis using the *cjpowR* package for the software R with *α* = 0.05 and *β* = 0.2. For details of the power analysis, see Supplementary Note 3.

The number of participants who began responding to the survey was 2061; 1066 completed the survey (female, 51.2%; age, mean = 48.6, SD = 16.51; respondents living in Tokyo, 12.6%). The sample did not deviate significantly from the population. The firm paid respondents in the form of redeemable points after the survey (the exact amount was not revealed to the authors). Participants who did not complete the survey included 856 who were screened out by an attention check on the first page, and 139 who dropped out of the survey. We also screened and excluded residents living abroad, healthcare workers/officials, journalists, and stakeholders with survey firms. The questionnaire consisted of five sections: socio-demographics; perceptions of COVID-19; conjoint experiment; associations with foreigners; and other related questions.

### Outcome variables

The outcome variables comprise three questions as given below. First, we asked question (a) to choose between two travelers, including an option of “Neither should be admitted”. Second, question (b) requested respondents to rate the degree to which they thought each traveler should be admitted. Lastly, question (c) was only for those who chose “Neither should be admitted” in the first question (33.1% of all tasks in total), and it had only two response options.

(a) Choice-based (3 options): If you have to choose between these two travelers, which one do you think should be admitted to enter Japan?

Traveler A/Traveler B/Neither should be admitted.

(b) Rating-based: What do you think about whether these travelers should be admitted to enter Japan, on a scale from 1 (definitely should not be admitted) to 7 (definitely should be admitted)?

Traveler A on a 7-point scale, recoded for analysis to take values from 0 to 1

Traveler B on a 7-point scale, recoded for analysis to take values from 0 to 1

(c) Choice-based (2 options), to respondents who chose “Neither should be admitted” to the first question: If you are forced to choose between these two travelers, which one do you think should be admitted to enter Japan?

Traveler A/Traveler B

### Subgroup analyses

Additionally, we planned to explore the impact of three significant factors associated with outgroup bias during a pandemic regarding host residents’ characteristics. Namely, people in the host country with higher risk perceptions of COVID-19, conservative partisanship, and weaker associations with foreigners would be less likely to admit entry to inbound travelers. To explore these factors, we asked seven questions for subgroup analyses to explore the heterogeneous effect of health certification by host residents’ characteristics as pre-registered: risk perceptions of COVID-19, associations with foreigners, and political partisanship. For details of the pre-registration, see Supplementary Note 2.

In terms of the risk perception of COVID-19, we measured three types of perceptions. *Infection Risk* was measured using the self-reported probability of the respondent contracting the infection within the next year (min = 0, max = 100, mean = 33.9, median = 30, SD = 20.5). We divided the respondents into “High” (*n* = 617), that is, those who answered higher than or equal to the median and “Low” (*n* = 449), that is, those who answered lower than the median. *Serious Risk* was measured using the self-reported probability of the respondent developing severe health conditions related to COVID-19 within the next year (min = 0, max = 100, mean = 22.5, median = 17, SD = 20.3). We divided respondents into “High” (*n* = 535), that is, those who responded higher than or equal to the median and “Low” (*n* = 531), that is, those who answered lower than the median. *Discomfort* was assessed to measure the emotional aspects of perceiving risks; following the studies on the BIS theory, we asked participants to rate, on a 7-point scale, their feelings when a person wearing a mask sneezed next to them (rescaled from 0 = “not at all uncomfortable” to 1 = “very uncomfortable”, mean = 0.55, median = 0.67, SD = 0.26). We divided the respondents into “High” (*n* = 558) for those who scored higher than or equal to the median and “Low” (*n* = 508) for those who scored lower.

To investigate the impact of associations with foreigners, we asked participants about contacts with foreigners and favorability for increasing number of foreigners in the country. *Foreigners’ Contacts* was measured using the first score by factor analysis regarding contacts with foreigners both during and before the COVID-19 pandemic on a 7-point scale (rescaled from 0 = “less than once per a few months” to 1 = “every day,” see Supplementary Table 2). We divided respondents into those who answered “Yes” to having contacts with foreigners (*n* = 368) and those who answered “No” (*n* = 698). *Foreigners’ Favorability* measured the extent to which the respondents favored increasing foreign workers and tourists on a 7-point scale (rescaled from 0 = “not favorable at all” to 1 = “very favorable”). Using the first score by factor analysis to integrate them (see Supplementary Table 2), we divided respondents into “High” (*n* = 544), that is, those who answered higher than or equal to the median and “Low” (*n* = 522), that is, those who answered lower than the median.

The questions to study political partisanship assessed party support and emotional temperature (i.e., grade of affinity). For *Conservative Support*, we categorized respondents into “Conservative” (*n* = 258), that is, those supporting the Liberal Democratic Party (LDP), Komeito, or the Japan Innovation Party (JIP), and “Others” (*n* = 783). LDP and Komeito formed a conservative coalition government from 1999 to 2009 and from 2012 to the present. JIP has taken a conservative position on an emblematic-ideological issue in Japan, reforming the Constitution of Japan, similar to LDP. *Conservative Temperature* measured the emotional aspects of partisanship by asking participants about their feelings using a thermometer with political parties and prime ministers leading the LDP–Komeito coalition government. Using the first score by factor analysis (see Supplementary Table 2), we divided respondents into “High” (*n* = 534) for those who scored higher than or equal to the median and “Low” (*n* = 532) for those who scored lower than the median.

### Estimation strategy

We employed linear regression models to examine hypotheses and estimated the marginal mean (MM) and uniform average marginal component effect (uAMCE). A classical work utilizing randomized conjoint analysis defined simple AMCE as representing “the marginal effect of attribute *l* averaged over the joint distribution of the remaining attributes.” (Hainmueller et al., 2014, p. 10) However, several scholars criticize AMCE for depending on the level used to estimate and propose utilizing MM to examine hypotheses, especially subgroup hypotheses (Clayton et al., 2021; Leeper et al., 2020). Cuesta, Egami, and Imai (2021) criticize that AMCE, which they call uAMCE, gives equal weight to all conjoint profiles. They propose the population average marginal component effect (pAMCE) using a real-world or counterfactual distribution considered theoretically.

**Fig. 1:**
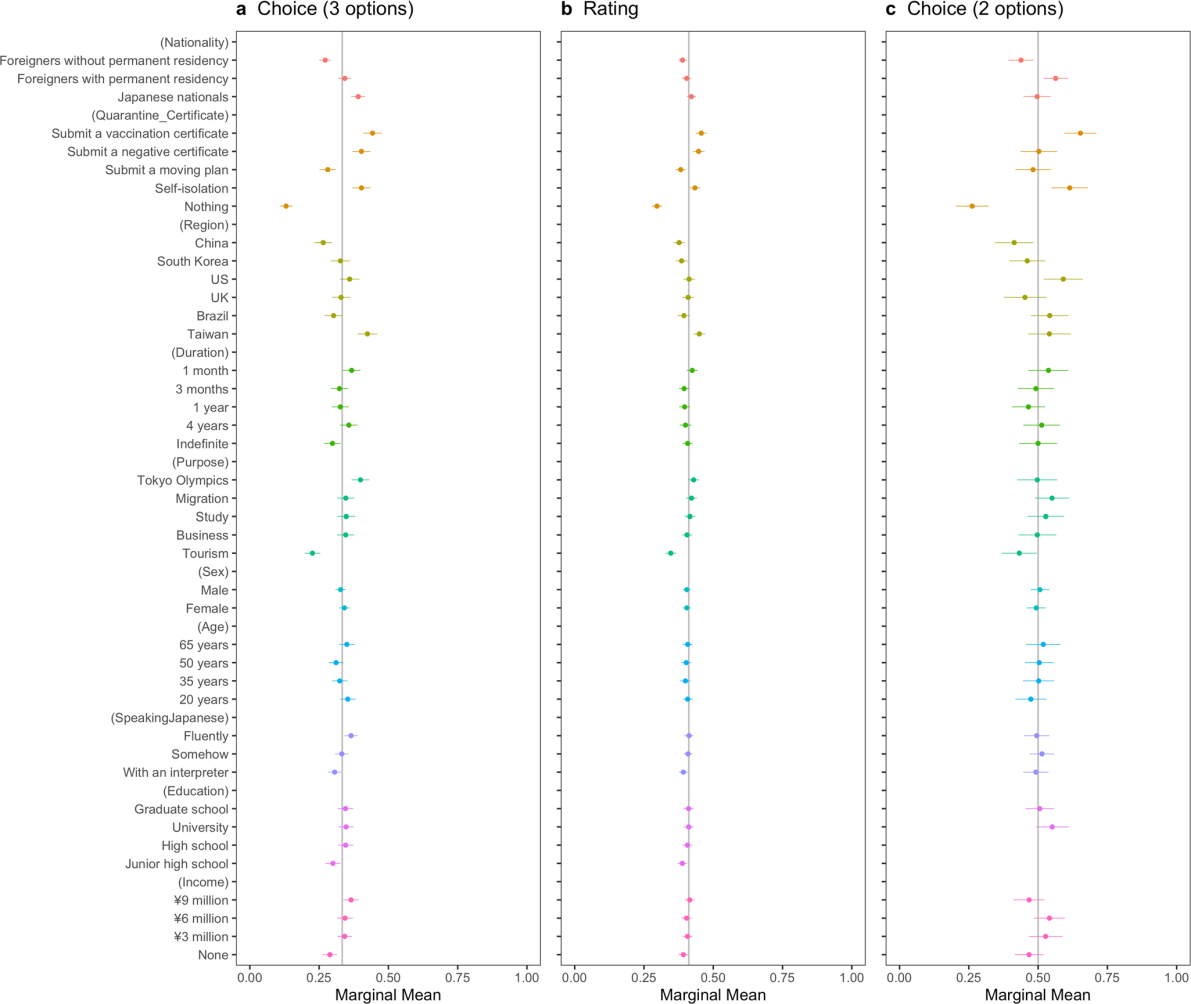
Marginal means of the respondents’ probability or rating of admitting travelers’ entry with error bars of 95 % confidence intervals. The vertical lines show the random probability (0.33) for Panel **a** using the choice-based question (three options including “Neither should be admitted”), the mean (0.41) for Panel **b** using the rating-based question, and the random probability (0.5) for Panel **c** using the choice-based question (two options).

**Fig. 2:**
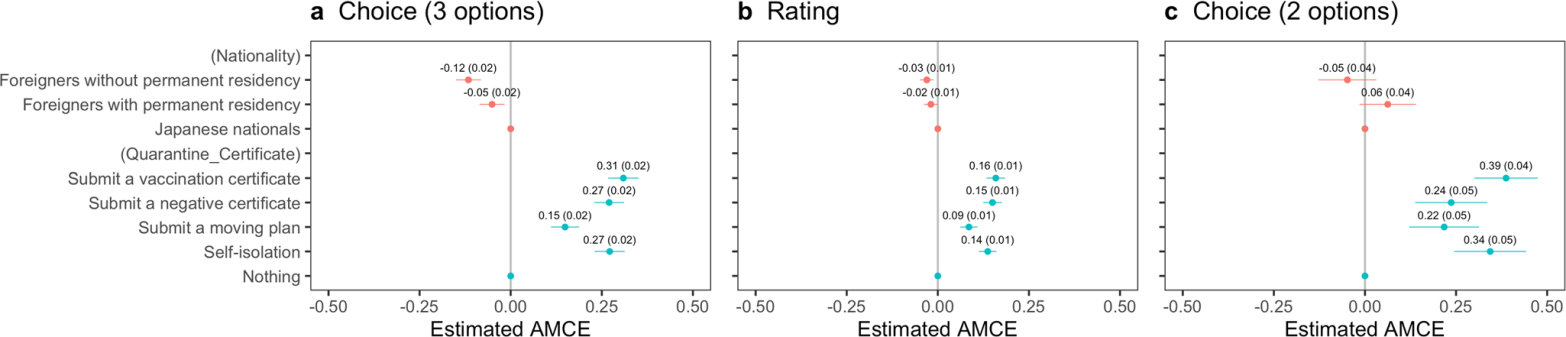
Uniform average marginal component effects of *Nationality* and *Quarantine_Certificate* on the probability or rating for travelers’ entry with error bars of 95% confidence intervals. The vertical lines (0.0) show no effects for Panel **a** using the choice-based question (three options including “Neither should be admitted”), Panel **b** using the rating-based question, and for Panel **c** using the choice-based question (two options).

**Fig. 3:**
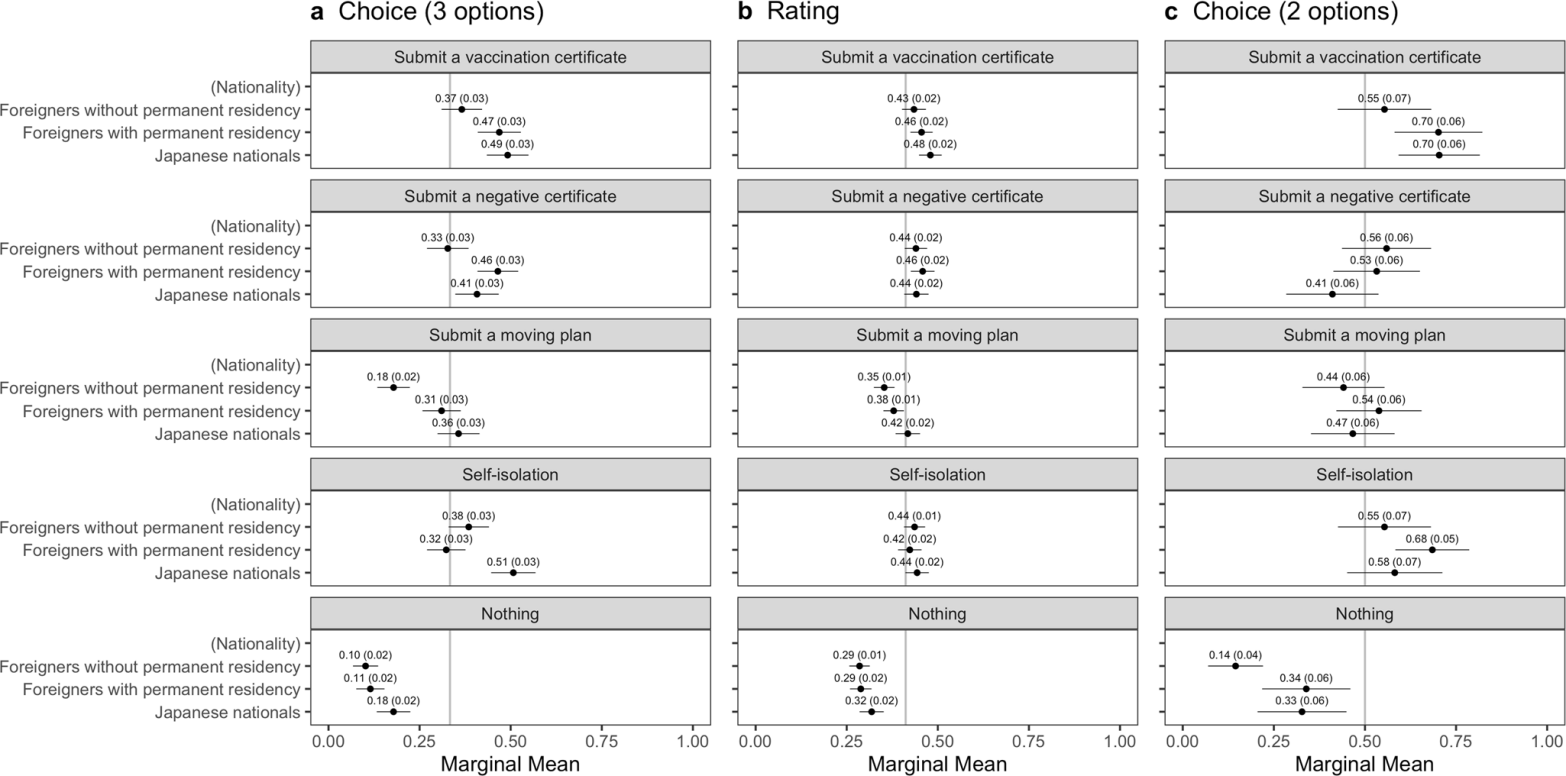
The interaction effects of *Quarantine_Certificate* with *Nationality* on the marginal means of the probability or rating for travelers’ entry with error bars of 95% confidence intervals. The vertical lines show the random probability (0.33) for Panel **a** using the choice-based question (three options including “Neither should be admitted”), the mean (0.41) for Panel **b** using the rating-based question, and the random probability (0.5) for Panel **c** using the choice-based question (two options).

**Fig. 4:**
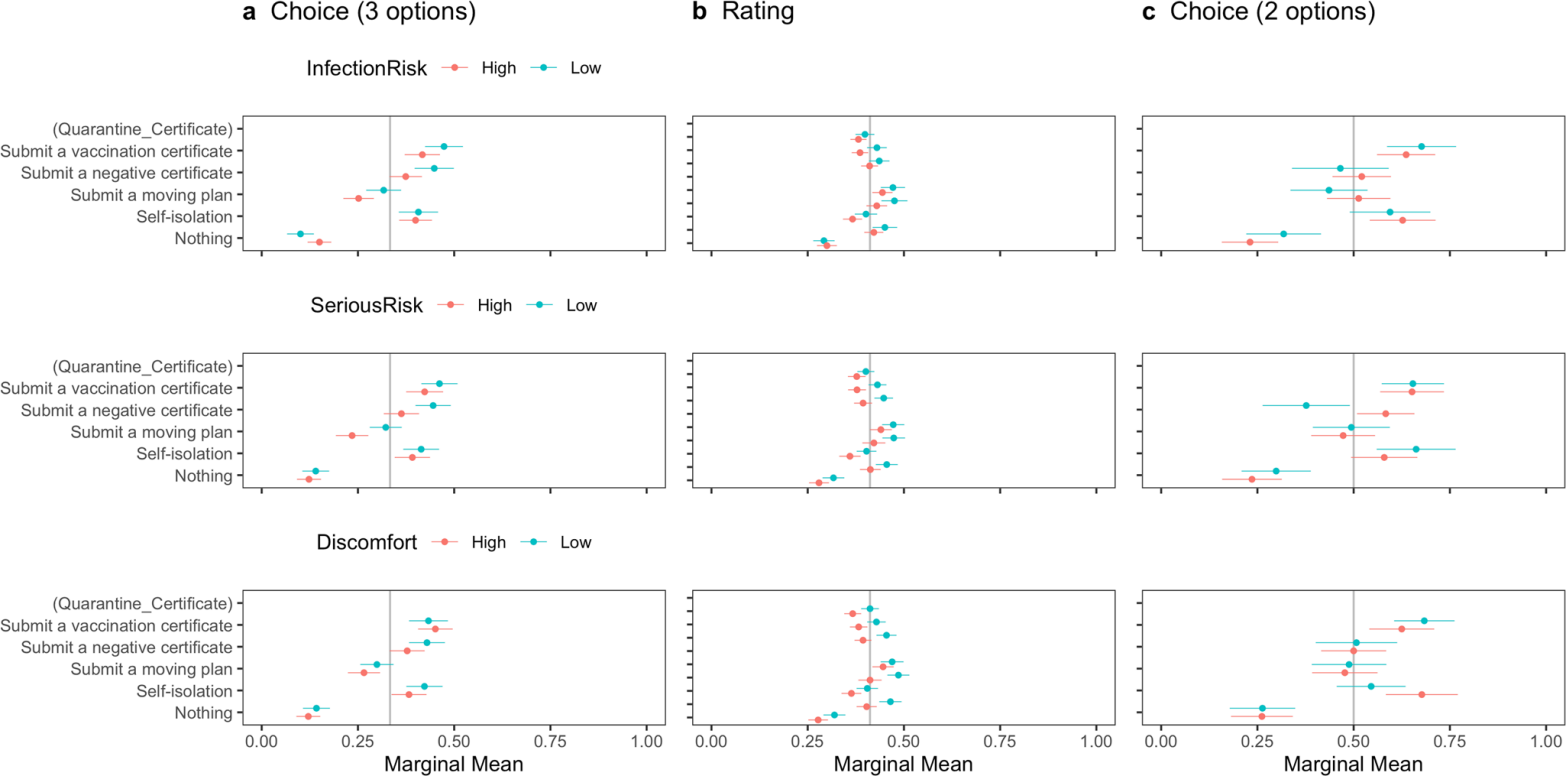
Marginal means of the probability or rating for travelers’ entry conditioned by risk perceptions of COVID-19 with error bars of 95% confidence intervals. The vertical lines show the random probability (0.33) for Panel **a** using the choice-based question (three options including “Neither should be admitted”), the mean (0.41) for Panel **b** using the rating-based question, and the random probability (0.5) for Panel **c** using the choice-based question (two options).

**Fig. 5:**
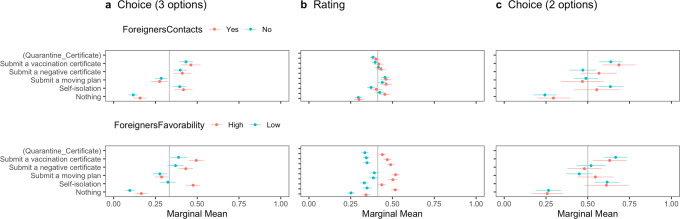
Marginal means of the probability or rating for travelers’ entry conditioned by associations with foreigners with error bars of 95% confidence intervals. The vertical lines show the random probability (0.33) for Panel **a** using the choice-based question (three options including “Neither should be admitted”), the mean (0.41) for Panel **b** using the rating-based question, and the random probability (0.5) for Panel **c** using the choice-based question (two options).

**Fig. 6:**
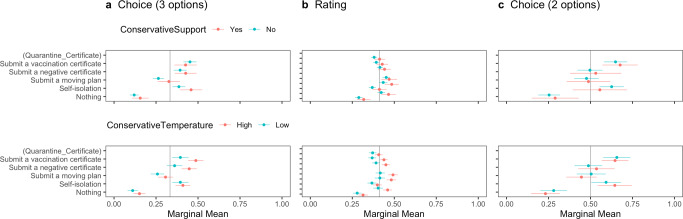
Marginal means of the probability or rating for travelers’ entry conditioned by conservative partisanship with error bars of 95% confidence intervals. The vertical line shows the random probability (0.33) for Panel **a**, the choice-based question (three options including “Neither should be admitted”), the mean (0.41) for Panel **b** using the rating-based question, and the random probability (0.5) for Panel **c**, the choice-based question (two options).

Following the methodological discussion, we first observed the difference in MMs of all attributes according to the pre-registered plan (Fig. 1). Second, we calculated the uAMCEs of significant attributes (Fig. 2), that is, *Nationality* and *Quarantine_Certificate*, followed by the MMs of those interaction (Fig. 3) to examine the effects of health certification in reducing outgroup bias. Lastly, to explore the heterogeneous effect of health certifications, we estimated MMs conditioned by host residents’ risk perceptions of COVID-19, associations with foreigners, and political partisanship (Figs. 4, 5, 6). All estimations were implemented by the *cregg* package (Leeper, 2020) in the software R. We used *p* < 0.05 criterion and two-tailed tests to examine the hypotheses, with standard errors clustered at the respondent level. We did not correct the p-values for testing multiple ideas as pre-registered. The script and data for the analyses are available via OSF 10.17605/OSF.IO/342UH.

## Results

### Estimating the effect of inbound travelers’ attributes

Figure 1 summarizes the MMs of probability and rating about admitting inbound travelers into Japan, based on the participants’ responses. The points indicate the means, while the whiskers show 95% confidential intervals under the condition that other things are equal. In any panel of Fig. 1, estimates to the left of the vertical lines indicate that respondents are less likely to admit the inbound travelers’ entry, whereas estimates to the right of the vertical lines indicate that respondents are more likely to admit the inbound travelers’ entry. The vertical line in Panel (a) indicates the random probability of the choice-based question with 3 options (i.e., 0.33). The vertical line in Panel (b) represents the mean of the rating-based question on a 7-point scale (i.e., 0.41). The vertical line in Panel (c) shows the random probability of the choice-based question with 2 options (i.e., 0.5). The more distance the 95% confidence interval of each level has from the vertical line, the more statistically strong the result is.

The attribute of *Nationality* indicates differences between Japanese nationals and foreigners. For example, Panels (a) and (b) show that Japanese are more likely to be allowed to enter than foreigners, even compared to foreigners with permanent resident status. Panel (a) reveals that respondents admitted foreigners with permanent residency almost the same as at random, while those without residency were admitted only 27% of the times. These results support the hypothesis in the pre-registration, which indicates the occurrence of outgroup bias during the COVID-19 pandemic. However, in Panel (c), foreigners with permanent residency are more likely to be admitted at 56%.

Regarding the effect of health certification, *Quarantine_Certificate* shows a larger difference between “Nothing” and other levels as compared to the effect of *Nationality*. For example, in Panel (a), the admission probability of a traveler submitting a vaccine certificate is the highest at 44%, followed by submitting a negative certificate or self-isolation; inbound travelers without specific quarantine were allowed entry only 10% of the times by the respondents. Panel (b) shows similar results, using a rating-based question. These results support the hypothesis in the pre-registration. Nonetheless, Panel (c) reports that submitting a negative certificate affects less than a vaccination certificate, using choice-based questions (2 options).

Reviewing the attribute of *Region*, travelers from Taiwan, which had not experienced any noticeable outbreak until the time of our survey, are most likely to be accepted by respondents as we expected in the pre-registration. Specifically, Taiwan in Panel (a) shows the highest probability at 42%, followed by other regions. Meanwhile, the admission probability of travelers from China is the lowest at 26%. Nonetheless, in Panel (c), using choice-based questions (2 options), travelers from the US are most likely to be admitted to enter at 59%.

The attribute of *Duration* shows that travelers who stay up to one month are more likely to be admitted than those who remain for an indefinite period. It is contrary to the expectation in the pre-registered plan, which assumed that respondents would be more likely to accept those with a longer stay. *Purpose* indicates a significant difference between travelers for the Tokyo Olympics and tourism. In Panel (a), respondents admitted 40% of travelers’ entry for the Tokyo Olympics, whereas 23% for tourism. Furthermore, *Speaking Japanese*, *Education*, and *Income* show significant differences in Panel (a). Inbound travelers with lower Japanese proficiency, lower educational level, or lower-income level are less likely to be admitted. However, those effects are insignificant in Panels (b) and (c).

### Estimating the effect of health certification in reducing outgroup bias

We examined the effect of *Nationality* and *Quarantine_Certificate*, followed by the effects of their interaction. Figure 2 estimates the uniform average marginal component effect (uAMCEs) of *Nationality* and *Quarantine_Certificate*. The estimates on the X-axis are the coefficients representing the rates of change compared to the baselines. The baseline for *Nationality* is “Japanese nationals”, and the baseline for *Quarantine_Certificate* is “Nothing”, that is, travelers with no quarantines. In Fig. 2, negative coefficients for a specific level indicate that respondents are less likely to admit entry to travelers, whereas a positive coefficient for a specific level indicates that respondents are more likely to allow entry, as compared to the baselines.

Regarding the attribute of *Nationalit*y, Panel (a) shows that respondents are 12% less likely to allow entry to foreign travelers without permanent residency than to Japanese nationals. Even if foreign travelers have permanent residency, they are 5% less likely to be allowed to enter the country than their Japanese counterparts. These results show the outgroup bias during the COVID-19 pandemic caused by travelers’ nationality as we presupposed.

Regarding the attribute of *Quarantine_Certificate*, in Panel (a), respondents are 27% more likely to admit travelers’ entry with self-isolation compared to those with no such quarantines or health certification. Importantly, offering health certification has almost the same effect as self-isolation on admission probability: travelers submitting a vaccination or negative certificate are more likely to be admitted at 31% and 27%, respectively. Panel (b), using the rating-based question with three options, shows similar results. Again, offering a vaccination or negative certificate has almost the same effect as self-isolation. However, using the choice-based question (2 options), Panel (c) reveals that only a vaccine certificate is as effective as self-isolation. In contrast, the result of submitting a negative certificate is only as low as offering a moving plan.

Here, we estimated the difference in the MMs of health certification interacting with travelers’ nationality or host residents’ characteristics to examine the effect of health certification in reducing outgroup bias among host residents. Figure 3 reports the MMs of *Quarantine_Certificate* by *Nationality*. This analysis investigated whether nationality can be a determinant between ingroups and outgroups. Indeed, Figs. 1 and 2 revealed that *Nationality* significantly affected the probability or rating of inbound travelers’ entry: Japanese were more likely and foreigners were less likely to be admitted to enter Japan by the respondents. Therefore, if health certification reduces outgroup bias, as we supposed, the difference between the same nationals and foreigners should decrease when the traveler submits a vaccination or negative certificate.

In Panels (a) and (b) of Fig. 3, while self-isolation raises the probability or rating of admitting only Japanese nationals, submitting a vaccination or negative certificate increases the probability of entry of the Japanese and foreigners with permanent residency. For example, looking at Panel (a), the admission probability of Japanese taking self-isolation is the highest at 51%, followed by Japanese submitting a vaccination certificate (49%), foreigners with permanent residency submitting a vaccination certificate (47%), those submitting a negative certificate (46%), and Japanese submitting a negative certificate (41%). Panel (c) reports the same effect of only vaccination certificates on admitting entry to Japanese and foreigners with permanent residency, but no effect of negative certificates.

### Exploring the heterogeneous effect of health certification by host residents’ characteristics

We also explored the difference in the MMs of health certification based on host residents’ characteristics to investigate the heterogeneity of the effect of health certification in reducing outgroup bias among host residents. We checked the differences according to respondents’ characteristics as pre-registered: risk perceptions of COVID-19, associations with foreigners, and political partisanship. The pre-registered hypotheses were that host residents’ higher risk perceptions, lower associations with foreigners, and political conservatism would negatively affect their attitudes towards inbound travelers (see Supplementary Note 2). Figures 4, 5, 6 report the subgroups’ conditional marginal means of *Quarantine_Certificate* for travelers’ entry.

Figure 4 shows that respondents’ risk perceptions of COVID-19 do not condition the impact of vaccination certificates while conditioning the effect of negative certificates on admitting travelers’ entry. In all the nine panels in Fig. 4, submitting a vaccination certificate does not significantly differ between any pair of subgroups. In contrast, offering a negative certificate makes a considerable difference between the two subgroups divided by *Serious Risk*, as shown in Panel (a) using the choice-based question (3 options), and *Discomfort*, as shown in Panel (b) using the rating-based question.

Figure 5 reports that respondents’ associations with foreigners do not condition the impact of health certification on admitting travelers’ entry. There is little difference between the two subgroups in the upper panels regarding *Foreigners’ Contacts*. In contrast, the bottoms of Panels (a) and (b) show that higher *Foreigners’ Favorability* produces a higher probability or rating of travelers’ entry irrespective of quarantine. However, we cannot find specific results on vaccination or negative certificates.

Figure 6 indicates that respondents’ conservative partisanship conditions the effect of health certification on admitting travelers’ entry. Regarding party support, the upper part in Panel (b), using the rating-based question, shows that conservative party supporters are more likely to permit entry of travelers submitting a negative certificate. Additionally, the bottoms in Panels (a) and (b) report that respondents having higher temperatures, namely, a higher grade of affinity, for conservative parties/politicians are more likely to admit entry by travelers submitting a vaccination or negative certificate.

## Discussion

Since the beginning of the COVID-19 pandemic, studies have reported evidence regarding outgroup bias worldwide against racial/ethnic groups, immigrants, and tourists. This study investigated the factors that reduce such outgroup bias using a discrete choice experiment with a randomized conjoint design in Japan, asking about inbound travelers’ entry, including foreigners, immigrants, and tourists. We found that travelers carrying health certification, that is, a vaccination or COVID-19 negative certificate, have a higher probability or rating of being admitted entry by host residents, with the same size as self-isolation. Significantly, health certification raises the chance or rating of foreigners with permanent residency almost up to the same admission level as that for Japanese nationals. Thus, our results demonstrate that health certification reduces outgroup bias among ingroup members facing threats to health by COVID-19.

This study not only reconfirms some previous claims by the BIS studies but also provides novel findings that can guide policy measures to mitigate outgroup bias. On the one hand, Figs. 1 and 2 reported a remarkable difference between the same nationals and foreign nationals in their chance or rating of being allowed to enter by host residents, a fact that ratifies other studies arguing outgroup bias during the COVID-19 pandemic. On the other hand, Fig. 3 supports our claim that health certification mitigates such outgroup bias between the same nationals and foreigners with permanent residency. Importantly, it is consistent with the studies on the BIS theory focused on social aspects, particularly an experimental finding that also exemplified that people avoid individuals with high pathogen risks despite group membership in the US and India (van Leeuwen and Petersen, 2018). This study suggests that policy interventions utilizing health certification can mitigate the outgroup bias predicted by the BIS.

We anticipate that the results of this study would facilitate the introduction of a vaccine passport and the usage of a negative certificate as an alternative to a vaccine passport. One of the issues in policy debates regarding vaccine passports has been a concern that they may cause inequality between vaccinated and unvaccinated people. However, such debates have underestimated the importance of psychological effects on the perception of ingroup members towards outgroup members regarding country or nationality and the impact of vaccine passports in reducing the bias. In contrast, the current study indicated that vaccine passports raise host residents’ acceptance of inbound travelers and promote equal preferability between nationals and foreigners having permanent resident status when entering the country. At the same time, a negative certificate is a potential alternative to a vaccination certificate since it has the same effect in raising the probability or rating of inbound travelers’ entry. In sum, this study provided evidence to introduce a combination of vaccine passports and negative certificates by shedding light on outgroup bias.

However, there was a considerable difference between vaccine certificates and negative certificates in reducing outgroup bias. The exploratory analyses on the heterogeneity across host residents’ characteristics showed that only the effect of a negative certificate in reducing outgroup bias was conditioned by higher risk perceptions of COVID-19, that is, *Serious Risk* and *Discomfort*. Likewise, conservative partisanship conditioned the impact of only a negative certificate in reducing outgroup bias. In other words, negative certificates had a less robust effect in reducing outgroup bias than vaccination certificates. Policymakers should consider that the effectiveness of negative certificates in mitigating outgroup bias depends on the social or political contexts of the country.

This study has three limitations, based on which we recommend future research agendas. First, we did not thoroughly examine the mechanism by which heterogeneous effects occur. For example, we omitted the role of trust in government, science, or the healthcare system, which would be associated with the impact of health certification in reducing outgroup bias. Additionally, border control policies in the months preceding our survey might have affected the attitudes of host residents toward inbound travelers. Second, our population-based experiment was conducted under a particular situation, in particular, under the second State of Emergency in Japan, while waiting to host the Tokyo Olympics/Paralympics. More longitudinal or cross-sectional studies are needed to verify the robustness of the results. Third, our conjoint design adopted a uniform distribution of levels, even though the causal inference using it may be inconsistent with a causal inference using a proportional distribution (Cuesta et al., 2021). The experimental results need to be verified by replicating the experiment or conducting a modified investigation.

## Conclusion

Despite these limitations, we revealed that health certification reduces the negative bias of host residents against inbound travelers in Japan during the COVID-19 pandemic. This study supposed that Japan could be a crucial case for countries to host mega international events. Health certification will be an essential tool for such countries to legitimize inbound travelers joining the event from abroad. However, the exceptional entries of participants in the Tokyo Olympics/Paralympics reduced the likelihood of entry from other travelers, including Japanese nationals. This was due to limited quarantine capacity at the airports (see Supplementary Note 1). Just as immigration policies are closely related to the status of immigrants at such domestic scenes as workplaces and social welfare offices (Bosworth and Guild, 2008; Cornelius, 2005), the readiness of medical capacities such as testing, monitoring, and implementing quarantines plays an important role in forming border policies during a pandemic. In that sense, health certification will also mitigate the quarantine burden to utilize the country’s limited capacity.

## Supplementary information

Supplementary Notes and Tables (Clean Version)

## Data Availability

The data for this study are available via OSF at 10.17605/OSF.IO/342UH.

## Code availability

All code for this study are available via OSF at 10.17605/OSF.IO/342UH.
