# Supplementary material for: COVID-19 health certification reduces outgroup bias: evidence from a conjoint experiment in Japan
Source: Humanit Soc Sci Commun. 2022 Sep 9;9(1):306. doi: 10.1057/s41599-022-01324-z (PMC9462644; doi:10.1057/s41599-022-01324-z)
Supplement: Supplementary file 1 — Supplementary Notes and Tables (Clean Version) [file 41599_2022_1324_MOESM1_ESM.docx]

**Supplementary information for “COVID-19 health certification reduces outgroup bias: evidence from a conjoint experiment in Japan”**

**Author information**

Yoshiaki Kubo

Department of Law, Politics, and International Relations, Faculty of Humanities and Social Sciences, University of the Ryukyus, Nishihara, Okinawa, Japan

Research Institute for Humanity and Nature, Kyoto, Japan

Department of East Asian Languages and Cultures, Hamilton Lugar School of Global and International Studies, Indiana University, Bloomington, IN, USA

Program on U.S.-Japan Relations, Weatherhead Center for International Affairs, Harvard University, Cambridge, MA, USA

Department of Urban Studies, School of Policy Studies, Kwansei Gakuin University, Sanda, Hyogo, Japan, present address

Isamu Okada

Department of International Development and Cooperation Studies, Graduate School of International Development, Nagoya University, Nagoya, Japan

**Corresponding author**

Yoshiaki Kubo

Email: kuboy@kwansei.ac.jp

Supplementary Notes 1-3

Supplementary Tables 1-2

Supplementary References

**Supplementary Notes**

**Supplementary Notes 1: The events related to this article in Japan under the COVID-19 pandemic.**

These notes overview the border controls by Japan under the COVID-19 pandemic. Information regarding governmental decisions on border control under this pandemic is accessible on the website of the headquarter<https://www.kantei.go.jp/jp/singi/novel_coronavirus/taisaku_honbu.html>. We accessed all the websites cited by this article on 13 May 2022.

The Japanese government policies aimed at reducing the chance of coronavirus intrusion into the country were selective and gradual and often criticized for being so. Two days after the World Health Organization declared a Public Health Emergency of International Concern on 30 January 2020, Japan closed its border to Chinese citizens with a passport issued in the Hubei province. By mid-February, passport-holders from the Zhejiang province were added. In the following weeks, selected areas within South Korea (mainly Daegu city) and Iran were included following the infection spread in each place. Regular quarantine checks were also ordered at major international airports. These restrictions targeting travelers from selected Asian areas were quick enough to contain the first intrusion at a minimal level.

During this early stage, Japan experienced three significant events domestically: the flight evacuation mission from Wuhan, the outbreak on the Diamond Princess (DP) cruise ship, and the nationwide school closure. In the flight evacuation mission, passengers were tested by the Polymerase Chain Reaction (PCR) method, self-isolation for 14 days, then retested before discharge (Kamata et al., 2020). In the case of DP, the government indicated whoever was either negative by PCR testing or was asymptomatic should disembark, then turned to set 14 days health observation period due to the high positive rate in initial cases (Mizumoto et al., 2020; Nakazawa et al., 2020). Lastly, Prime Minister Shinzo Abe requested the nationwide school closure on 27 February, then conducted from 2 March, which continued to the start of the next academic year in April 2020 (Fukumoto et al., 2021).

While these initial responses, the epidemiologists who assisted the Ministry of Health, Labour and Welfare (MHLW) stayed cautious of the growing numbers of imported cases from Europe, Southeast Asia, and Egypt. About the perceptions of the experts working closely with the governments, see the report by the Independent Investigation Commission on the Japanese Government’s Response to COVID-19, English version<https://apinitiative.org/wp/wp-content/uploads/2021/01/2-9_API-Independent-Investigation-Commission-on-Japanese-Response-to-COVID.pdf>).

On 17 March, the very next day of EU’s border closure across its member countries and from abroad, the panel of Japanese experts submitted a petition to the government urging a stricter border control. Two days later, the government headquarter for COVID-19 reluctantly closed the border to selected areas in Italy, Switzerland, Spain, and Iceland. It took another week to entirely ban a new entry from major EU countries. Finally, the headquarters authorized the border closure for any passengers from China, South Korea, the US, the UK, Brazil, and other 44 countries as late as 1 April 2020, a day after the decision to postpone the Tokyo Olympics/Paralympics. These closures restricted not just entry by new visitors such as tourists but also re-entry by foreigners with permanent or temporary residency who had left Japan after the enforcement day, 3 April. Yet, the infected number reached its first peak in mid-April, and the government declared the first State of Emergency between 7 April and 25 May.

One major criticism targeted the ostensibly delayed decision-making within the government. In a report by civil society volunteers who organized an independent investigation commission and interviewed the stakeholders involved in the headquarter, three reasons occasioned the sluggishness (API, 2020). First, border control was an unprecedented policy issue that involved several Ministries. However, the coordination problem was resolved when the Prime Minister commissioned the National Security Secretariat to lead the process. Second, multiple—sometimes conflicting—interests were involved. In early 2020, two significant events were apparently within the scope of the government: the visit of the Chinese President to Japan, not realized since 2008, and the Tokyo Olympics/Paralympics. Postponing the visit of the Chinese authority was decided on 5 March 2020, the same day that Japanese headquarter agreed to introduce strict quarantine to all travelers from China and South Korea. The postponement of the Olympics/Paralympics coincided with the border closure for 45 countries, as previously mentioned. Third, negative prospects against any stringent top-down measures had become a dominant voice within the government after the unpopular school closure from the end of February (Takaku and Yokoyama, 2021). In all cases, the politicians seemed to gauge how border restriction would affect them politically.

From July to December 2020, the Japanese government gradually shifted from a total ban to reopening, although the infected number reached the second peak in early August. The process relied on infection trends in Japan and corresponding countries and upgraded surveillance capacity in the public sector. In addition, following the relaxed international movement for business travelers between China and South Korea in May 2020, Japan sought to establish the criteria for selective reopening to selected countries. As a result, foreign workers and technical trainees for a long-term stay with 14 days of self-isolation were permitted for Thailand and Vietnam (29 July), Taiwan, Malaysia, Cambodia, Laos, Myanmar (8 September), Singapore (30 September), South Korea, and Brunei (8 October), and China (30 November). Also, the government lifted the restriction on the re-entry of foreigners with a permanent or temporary residency on 1 September 2020.

The subsequent period from October to December 2020 coincided with the campaigns of new Prime Minister Yoshihide Suga, who proclaimed economic recovery after containing the second outbreak in August. Business travelers whose stay in Japan doesn’t exceed 72 hours were also allowed under the condition of on-time testing and without 14 days of self-isolation, for Singapore (18 September), South Korea (8 October), Vietnam (1 November), and China (30 November). Regardless of these specific tracks for selected nationals, all nationals were accepted for a new entry from 1 October 2020, provided that they follow 14 days self-isolation and other preemptive regulations. Furthermore, the government started the Olympic and Paralympic Preparation Track for Athletes (shortly, Athlete Track) on 12 November. It allowed athletes, coaches, and staff of related games to join competitions or their work related to the Olympics/Paralympics without the 14-day waiting period after their entry as required elsewhere (TOCOPG, 2020).

However, this temporal and selective emancipation veered again toward a full closure since the end of December 2020. This change was mainly due to re-growing infection within Japan and abroad associated with new variants that caused outbreaks in other countries such as the UK. Responding to the infection increase, which reached the third peak in mid-January 2021, the government declared the second State of Emergency between 8 January and 21 March. Consequently, all the above-mentioned selective tracks were suspended since mid-January, while the re-entry of foreigners with permanent or temporal residency was continued to be permitted. Under this situation, we conducted the survey used in the article from 22 to 24 February 2021.

After ending the second State of Emergency, entry permits for people related to the Tokyo Olympics/Paralympics were resumed in late March. See Prime Minister Suga’s answer to a question from Yuichiro Tamaki, the Democratic Party for the People in the Committee on Rules and Administration, the House of Representatives, 204th Diet, 18 March 2021. It continued despite the third State of Emergency from 25 April to 20 June. However, the Olympics/Paralympics were held without the audience in principle from July to August. A reason for it was that the government declared another State of Emergency in the area, including Tokyo, on 12 July (the State of Emergency had been continued in Okinawa from 23 May). That was a response to the infection spreading of the delta variant. The fourth State of Emergency extended to other regions nationwide, then ended on 30 September 2021.

On 26 July, during the Olympics, the Japanese government started issuing vaccination certificates for outbound travelers. Nonetheless, it was 1 October 2021 when Japan began using vaccination certificates to ease inbound travelers’ 14 days of self-isolation after the entry.

**Supplementary Note 2: The hypotheses in the pre-registration**

We planned to examine what types of travelers from abroad were less likely to be admitted by what type of people in the host country. A traveler was a category to describe people who travel regardless of purpose (e.g., tourism, business, study, or immigration). Thus, we built hypotheses regarding travelers (H1-4) and host residents (H5-7). Those are modified from the original version <https://doi.org/10.17605/OSF.IO/MHKZ2> to clarify the meaning, but we did not change the basic ideas.

Regarding travelers’ characteristics, we predicted that people in the host country would be less likely to admit entry to inbound travelers having foreign nationality (H1), from infection-spread regions (H2), for a short stay (H3), and with inadequate quarantines or without a proper health certificate (H4).

H1 was induced from psychological theories, i.e., terror management theory (TMT), integrated/intergroup threat theory (ITT), system justification theory (SJT), and behavioral immune system (BIS) theory, as reviewed previously in the Main. Those have argued that infectious threats reinforce outgroup bias due to outgroup derogation or ingroup favoritism. Therefore, as inbound foreign travelers are a typical outgroup for host residents, we predicted that host residents would admit less inbound entry to travelers having foreign nationality (H1).

H2-4 is induced from the behavioral immune system (BIS) theory. Against the traditional account for BIS that the system is motivated to avoid correlates of pathogens, a study argued that it was motivated to prevent individuals with high pathogen risks despite group membership (van Leeuwen and Petersen, 2018). Indeed, it found evidence supporting the argument through experiments in the US and India. This argument is also consistent with social psychological perspectives, i.e., reducing anxiety/uncertainty from threats to life (TMT), group (ITT), or system (SJT) increases outgroup bias. For example, a field experiment in India reported that accurate and focused information on COVID-19 decreased the chance of stigmatizing patients, including foreign nationals (Islam et al., 2021). Hence, we hypothesized that travelers with growing anxiety for host residents about the pathogen, such as from widespread regions (H2), staying shortly (H3), or with inadequate quarantines or without a proper health certificate (H4), would be less likely to be admitted entry.

Furthermore, we planned to explore the impact of three significant factors associated with outgroup bias under pandemics regarding host residents' characteristics. Namely, people in the host country with higher risk perceptions of COVID-19 (H5), conservative partisanship (H6), and weaker associations with foreigners (H7) would be less likely to admit entry to inbound travelers.

H5 was induced from two assumptions. First, infection risk perceptions for host residents depend on their characteristics, such as pre-existing conditions, living with persons vulnerable to diseases, and living in a wide infection-spreading area. Second, higher risk perceptions cause higher feelings of threat. For example, a survey in Italy reported risk perceptions of Ebola were positively associated with prejudice toward immigrants (Prati and Pietrantoni, 2016). In addition, an experiment in the US said people with a higher perceived vulnerability to disease set a lower threshold for categorizing targets as disease cues (Miller and Maner, 2012). Thus, we predicted that host residents with higher risk perceptions of COVID-19 would be less likely to admit inbound travelers (H5).

H6 was induced from two assumptions: (1) perceiving threats is strongly associated with political conservatism, and; (2) partisanship or political ideology is associated with outgroup attitudes. Meta-analyses in social psychology (Jost et al., 2003; Terrizzi et al., 2013) support the first, and several studies (on immigrants (Aarøe et al., 2017), and refugees (Getmansky et al., 2018)) back the second. Notably, we assumed that political conservativeness conditions the effect of threats on attitudes toward foreigners. For example, a vignette experiment in the US found that when Republican citizens read a statement by Republican politicians criticizing responses to Ebola by the Obama administration, attitudes toward immigrants were negatively biased (Adida et al., 2020). Hence, we predicted that host residents with a specific ideology, such as conservative in the US, would be less likely to admit inbound travelers (H6).

Lastly, H7 was induced from two assumptions: (1) contact with outgroups causes reducing prejudice against them, and; (2) its effect is mediated by enhancing knowledge about the outgroup, reducing anxiety about contact, and increasing empathy and perspective-taking. Both were supported by meta-analyses in social psychology (Pettigrew et al., 2011; Pettigrew and Tropp, 2008, 2006). Significantly, social relationships with and normative attitudes regarding outgroups affect the attitudes toward them. An experimental study based on the behavioral immune system theory revealed that people tend to avoid immigrants because they don’t believe they share local norms (Karinen et al., 2019). It implied normative aspects affect how they perceive inbound travelers. Therefore, we predicted that host residents having weak associations (i.e., fewer contacts or negative attitudes) with foreigners would be less likely to admit entry to inbound travelers (H7).

**Supplementary Note 3: Power analysis to decide the sample size.**

The survey planned to collect 4,000 observations (=1,000 respondents × 2 profiles × 2 tasks). We conducted a power analysis at a=0.05 and b=0.2 using the *cjpowR* package for the software R. It has several functions to decide the sample size of conjoint experiments with the number of levels and expected causal effects. The interest of the current study is the effect of health certification and its interaction with travelers' nationality on the probability of admitting entry by host residents. In our conjoint experiment, the attribute of *Quarantine_Certificate* had five levels, the attribute of *Nationality* had three levels, and the choice-based outcome variable had three options. To conduct the power analysis, we set 0.87 as the expected AMCE, i.e., 0.174 as the interaction coefficient, to identify a larger effect size because the 25 percentile of the average marginal component effects (AMCEs) was 0.02, the median was 0.05, and the 75 percentile was 0.87 for 15 articles with conjoint experiments highly cited (Schuessler and Freitag, 2020). With these conditions, the power analysis using the *cjpowr_amcie* function resulted in a required sample size of 3,564.

**Supplementary Tables**

**Supplementary Table 1: Translated attributes and levels of the conjoint design into English.**

|  | Attributes | Levels |
| --- | --- | --- |
| 1 | *Nationality* | Foreigners without permanent residency, Foreigners with permanent residency, Japanese nationals |
| 2 | *Quarantine_Certificate* | Submit a vaccination certificate, Submit a negative certificate, Submit a moving plan, Self-isolation, Nothing |
| 3 | *Region* | China, South Korea, the US, the UK, Brazil, Taiwan |
| 4 | *Duration* | 1 month, 3 months, 1 year, 4 years, Indefinite |
| 5 | *Purpose* | Tourism, Business, Study, Migration, Tokyo Olympics |
| 6 | *Sex* | Female, Male |
| 7 | *Age* | 20 years, 35 years, 50 years, 65 years |
| 8 | *Speaking Japanese* | With an interpreter, Somehow, Fluently |
| 9 | *Education* | Junior high school, High school, University, Graduate school |
| 10 | *Income* | None, ¥3 million, ¥6 million, ¥9 million |
| Note: Baselines in estimating uAMCE are underlined. In the experiment, two profiles were formulated by two types of randomization. First, the levels of attributes were randomly combined to create hypothetical traveler profiles. Second, the order of attributes was randomly displayed, except for these combinations of attributes: *Nationality*, *Region*, *Purpose*, *Duration*, *Quarantine_Certificate*; and *Gender*, *Age*, *Speaking Japanese*, *Education*, *Income*. To conduct the randomization, we used Qualtrics and Conjoint Survey Design Tool<https://github.com/astrezhnev/conjointsdt>, referring to the website of Dr. Jaehyun Song< https://www.jaysong.net/> in Japanese. | | |

**Supplementary Table 2: Factor analyses of associations with foreigners and emotional temperature.**

Contacts with foreigners:

|  | Factor loadings |
| --- | --- |
| At present (mean=.05, median=0, SD=.17) | .707 |
| Before the spreading of COVID-19 (mean=.07, median=0, SD=.21) | .707 |

Favorability about foreigners:

|  | Factor loadings |
| --- | --- |
| The number of foreign workers in Japan recorded the highest in 2019 (mean=.52, median=.5, SD=.23) | .707 |
| The number of foreign tourists visiting Japan recorded the highest in 2019 (mean=.62, median=.67, SD=.25) | .707 |

The emotional temperature on conservative parties/politicians (min=0, max=100):

|  | Factor loadings |
| --- | --- |
| The Liberal Democratic Party (mean=44.6, median=50, SD=22.7) | .563 |
| The Japan Communist Party (mean=31.4, median=30, SD=22.3) | -.207 |
| Yoshihide Suga (mean=40.9, median=50, SD=22.7) | .560 |
| Shinzo Abe (mean=41.3, median=49, SD=26.5) | .571 |

*Missing values were imputed by the mean.

**Supplementary References**

Aarøe, L., Petersen, M.B., Arceneaux, K., 2017. The behavioral immune system shapes political intuitions: Why and how individual differences in disgust sensitivity underlie opposition to immigration. Am. Polit. Sci. Rev. 111, 277–294. https://doi.org/10.1017/S0003055416000770

Adida, C.L., Dionne, K.Y., R. Platas, M., 2020. Ebola, elections, and immigration: How politicizing an epidemic can shape public attitudes. Polit. Groups Identities 8, 488–514. https://doi.org/10.1080/21565503.2018.1484376

API (Asia Pacific Initiative), 2020. The independent investigation commission on the Japanese Government’s response to COVID-19. AP Initiat. https://apinitiative.org/en/project/covid19/ (accessed 17 Jun 2021).

Fukumoto, K., McClean, C.T., Nakagawa, K., 2021. No causal effect of school closures in Japan on the spread of COVID-19 in spring 2020. Nat. Med. 1–9. https://doi.org/10.1038/s41591-021-01571-8

Getmansky, A., Sınmazdemir, T., Zeitzoff, T., 2018. Refugees, xenophobia, and domestic conflict: Evidence from a survey experiment in Turkey. J. Peace Res. 55, 491–507.

Islam, A., Pakrashi, D., Vlassopoulos, M., Wang, L.C., 2021. Stigma and misconceptions in the time of the COVID-19 pandemic: A field experiment in India. Soc. Sci. Med. 278, 113966. https://doi.org/10.1016/j.socscimed.2021.113966

Jost, J.T., Glaser, J., Kruglanski, A.W., Sulloway, F.J., 2003. Political conservatism as motivated social cognition. Psychol. Bull. 129, 339–375. https://doi.org/10.1037/0033-2909.129.3.339

Kamata, K., Jindai, K., Ide, K., Funaki, T., Saito, H., Takeshita, N., Ohmagari, N., Hinoshita, E., Asanuma, K., 2020. The flight evacuation mission for COVID-19 from Wuhan, China to Tokyo, Japan from 28 January to 17 February 2020. Jpn. J. Infect. Dis. advpub. https://doi.org/10.7883/yoken.JJID.2020.938

Karinen, A.K., Molho, C., Kupfer, T.R., Tybur, J.M., 2019. Disgust sensitivity and opposition to immigration: Does contact avoidance or resistance to foreign norms explain the relationship? J. Exp. Soc. Psychol. 84, 103817. https://doi.org/10.1016/j.jesp.2019.103817

Miller, S.L., Maner, J.K., 2012. Overperceiving disease cues: The basic cognition of the behavioral immune system. J. Pers. Soc. Psychol. 102, 1198–1213. https://doi.org/10.1037/a0027198

Mizumoto, K., Kagaya, K., Zarebski, A., Chowell, G., 2020. Estimating the asymptomatic proportion of coronavirus disease 2019 (COVID-19) cases on board the Diamond Princess cruise ship, Yokohama, Japan, 2020. Eurosurveillance 25, 2000180. https://doi.org/10.2807/1560-7917.ES.2020.25.10.2000180

Nakazawa, E., Ino, H., Akabayashi, A., 2020. Chronology of COVID-19 cases on the Diamond Princess cruise ship and ethical considerations: A report from Japan. Disaster Med. Public Health Prep. 14, 506–513. https://doi.org/10.1017/dmp.2020.50

Pettigrew, T.F., Tropp, L.R., 2008. How does intergroup contact reduce prejudice? Meta-analytic tests of three mediators. Eur. J. Soc. Psychol. 38, 922–934. https://doi.org/10.1002/ejsp.504

Pettigrew, T.F., Tropp, L.R., 2006. A meta-analytic test of intergroup contact theory. J. Pers. Soc. Psychol. 90, 751–783. https://doi.org/10.1037/0022-3514.90.5.751

Pettigrew, T.F., Tropp, L.R., Wagner, U., Christ, O., 2011. Recent advances in intergroup contact theory. Int. J. Intercult. Relat. 35, 271–280. https://doi.org/10.1016/j.ijintrel.2011.03.001

Prati, G., Pietrantoni, L., 2016. Knowledge, risk perceptions, and xenophobic attitudes: Evidence from Italy during the Ebola outbreak. Risk Anal. 36, 2000–2010. https://doi.org/10.1111/risa.12537

Schuessler, J., Freitag, M., 2020. Power analysis for conjoint experiments. https://doi.org/10.31235/osf.io/9yuhp

Takaku, R., Yokoyama, I., 2021. What the COVID-19 school closure left in its wake: Evidence from a regression discontinuity analysis in Japan. J. Public Econ. 195, 104364. https://doi.org/10.1016/j.jpubeco.2020.104364

Terrizzi, J.A., Shook, N.J., McDaniel, M.A., 2013. The behavioral immune system and social conservatism: A meta-analysis. Evol. Hum. Behav. 34, 99–108. https://doi.org/10.1016/j.evolhumbehav.2012.10.003

TOCOPG (the Tokyo Organising Committee of the Olympic and Paralympic Games), 2020. Fifth coordination meeting for COVID-19 countermeasures at the Olympic and Paralympic Games Tokyo 2020. https://olympics.com/tokyo-2020/en/news/fifth-coordination-meeting-for-covid-19-countermeasures-at-tokyo-2020 (accessed 15 Jun 2021).

van Leeuwen, F., Petersen, M.B., 2018. The behavioral immune system is designed to avoid infected individuals, not outgroups. Evol. Hum. Behav. 39, 226–234. https://doi.org/10.1016/j.evolhumbehav.2017.12.003
